# Supplementary material for: Nicotiana noctiflora Hook. Genome Contains Two Cellular T-DNAs with Functional Genes
Source: Plants (Basel). 2023 Nov 7;12(22):3787. doi: 10.3390/plants12223787 (PMC10674353; doi:10.3390/plants12223787)
Supplement: Supplementary file 1 [file plants-12-03787-s001.zip › plants-2633425-supplementary.pdf]

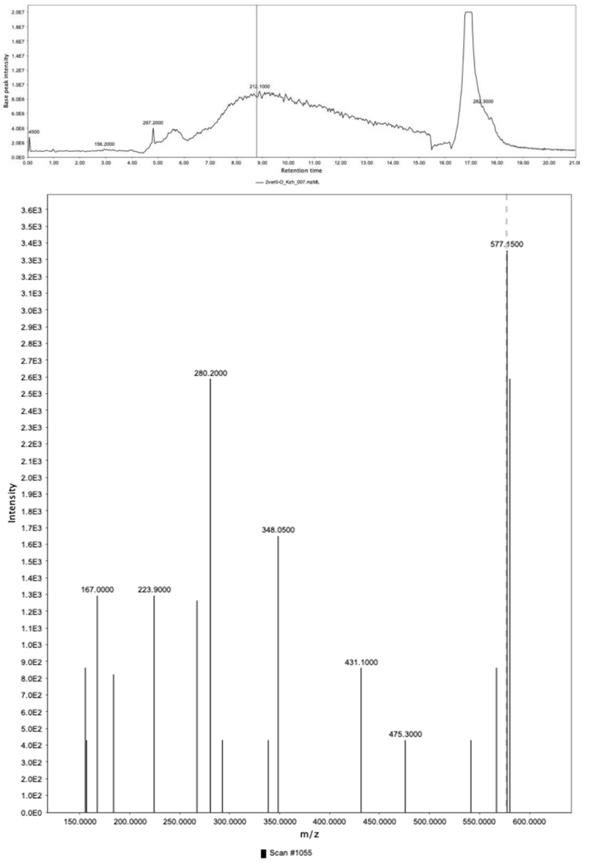

Figure S1 High performance liquid chromatography (HPLC) profile of agrocinopine-enriched extract HPLC-MS was performed on a Shimadzu LCMS-IT-TOF instrument according to Padilla et al (2021) [55]. The mobile phase was a mixture of acetonitrile and acidified water (0.4% formic acid) with a flow rate of 0.3 mL/min. The gradient started with 100% acidified water for 3 min, and then increased linearly to 100% acetonitrile over 4 min, maintained for 1 min before returning to the initial conditions, and then held to equilibrate the column after collection (2 min). The injection volume of each extract or standard solution was 5 µL. Electrospray ionization was used in positive and negative ionization modes, drying gas (N_2) flow 1.5 l/min. Detection was carried out in the following voltage range: -2.5, -1.5, -1, +1.5, +2, +3 kV.

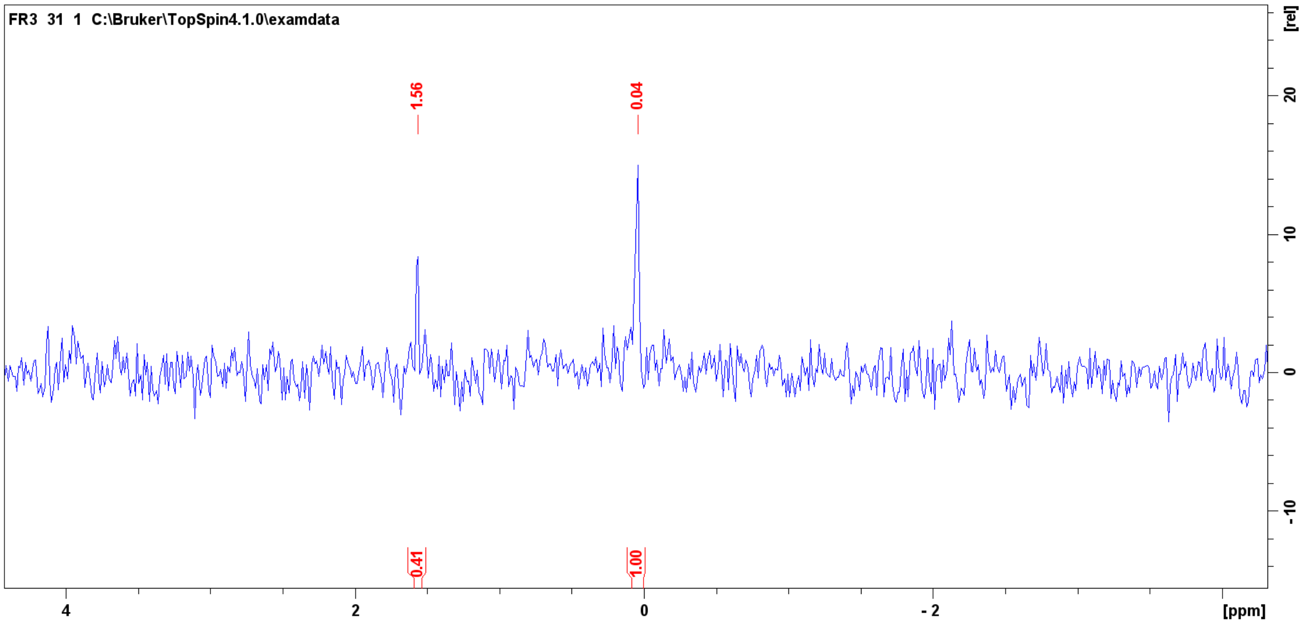

Figure S2. ^{31}P NMR spectra of agrocinopine-like structure

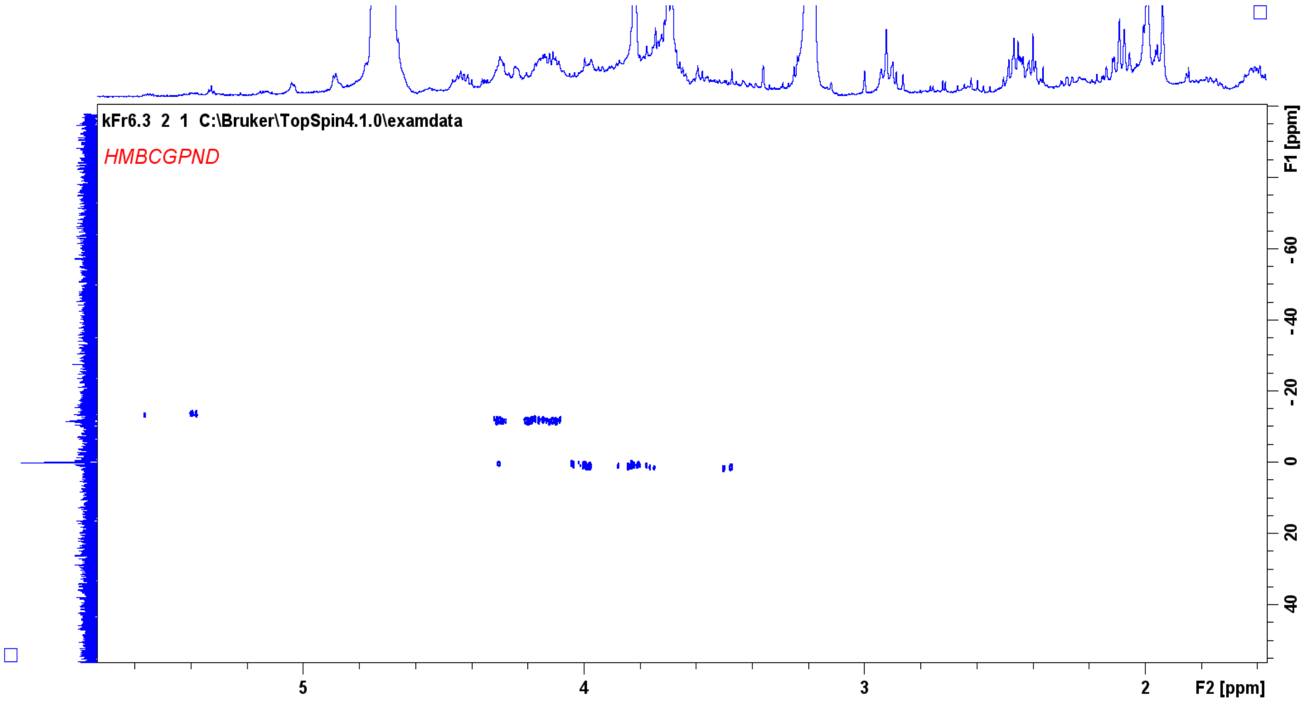

Figure S3. The 2D HMBC ^{1}H, ^{31}P spectra of agrocinopine-like structure
